# Metabolic plasticity in a *Pde6b*^*STOP/STOP*^ retinitis pigmentosa mouse model following rescue

**DOI:** 10.1016/j.molmet.2024.101994

**Published:** 2024-07-19

**Authors:** Monika Ayten, Nundehui Díaz-Lezama, Hanaa Ghanawi, Felia C. Haffelder, Jacqueline Kajtna, Tobias Straub, Marco Borso, Axel Imhof, Stefanie M. Hauck, Susanne F. Koch

**Affiliations:** 1 Department of Pharmacy, Center for Drug Research, Ludwig-Maximilians-Universität München, Munich, Germany; 2 Bioinformatics Unit, Biomedical Center Munich, Ludwig-Maximilians-Universität München, Munich, Germany; 3 Molecular Biology, Biomedical Center Munich, Faculty of Medicine, Ludwig-Maximilians-Universität München, Munich, Germany; 4 Metabolomics and Proteomics Core, Helmholtz Zentrum München, German Research Center for Environmental Health, Neuherberg, Germany

**Keywords:** Gene therapy, Retina, Retinitis pigmentosa, Retinal plasticity, Phototransduction, Inflammation, Metabolism, OXPHOS, Proteomics

## Abstract

**Objective:**

Retinitis pigmentosa (RP) is a hereditary retinal disease characterized by progressive photoreceptor degeneration, leading to vision loss. The best hope for a cure for RP lies in gene therapy. However, given that RP patients are most often diagnosed in the midst of ongoing photoreceptor degeneration, it is unknown how the retinal proteome changes as RP disease progresses, and which changes can be prevented, halted, or reversed by gene therapy.

**Methods:**

Here, we used a *Pde6b*-deficient RP gene therapy mouse model and performed untargeted proteomic analysis to identify changes in protein expression during degeneration and after treatment.

**Results:**

We demonstrated that *Pde6b* gene restoration led to a novel form of homeostatic plasticity in rod phototransduction which functionally compensates for the decreased number of rods. By profiling protein levels of metabolic genes and measuring metabolites, we observed an upregulation of proteins associated with oxidative phosphorylation in mutant and treated photoreceptors.

**Conclusion:**

In conclusion, the metabolic demands of the retina differ in our *Pde6b*-deficient RP mouse model and are not rescued by gene therapy treatment. These findings provide novel insights into features of both RP disease progression and long-term rescue with gene therapy.

## Highlights

• Genetic restoration of *Pde6b* rescued the expression of proteins involved in phototransduction and visual function.
• Degenerating retinas displayed enhanced expression in oxidative phosphorylation proteins and increased ATP production.
• Genetic restoration of *Pde6b* cannot halt changes in retinal metabolic reprogramming.

## 1 Introduction

Retinitis pigmentosa (RP) is the most prevalent inherited retinal disease worldwide, typically manifesting in adolescence or early adulthood [1]. Patients initially experience night blindness, followed by a gradual narrowing of the visual field, ultimately leading to the loss of daylight vision [2,3]. RP occurs primarily as a monogenic disease, with the underlying causative gene expressed in rod photoreceptor or retinal pigment epithelium (RPE) cells. This leads to progressive degeneration of rods, followed by secondary loss of cone photoreceptors, along with retinal remodeling, gliosis and inflammation [[4], [5], [6]]. To date, more than 90 different genes have been linked to RP, and gene therapy represents the most promising treatment strategy [3,[7], [8], [9]]. There is an FDA-approved gene therapy (Luxturna®) for a retinal degenerative disease caused by mutations in the RPE65 gene [8,9]. However, data from clinical trials raise sustainability concerns regarding adverse side effects and the long-term efficacy of Luxturna® treatment [10,11]. Notably, retinal atrophy has been described following treatment with Luxturna®, even in patients with substantial rod rescue [11,12]. Proposed limitations include the delivery method (subretinal injections), inflammatory responses, or changes in metabolic pathways [[13], [14], [15]]. Here, we used our RP gene therapy mouse model (*Pde6b*^*STOP/STOP*^*, Pde6g*^*CreERT2/*+^) which harbors a floxed stop cassette in both *Pde6b* alleles, preventing PDE6B expression in the absence of Cre recombinase activity [16]. After tamoxifen injection, *Pde6g*^*CreERT2*^ recombinase is activated, excising the stop cassette and leading to PDE6B expression. This Cre-driven *Pde6b* gene restoration model greatly facilitates the study of gene therapy limitations because it represents an idealized gene therapy scenario, where almost all rods are rescued. Moreover, it does not involve subretinal injections, the current method of therapeutic gene delivery (in humans and animals), which can lead to retinal detachment, cataracts, infection, bleeding, and trauma [17].

## 2 Materials and methods

### 2.1 Animals

All animal experiments were performed in accordance with the ARVO statement on the use of animals in ophthalmic and vision research and were approved by the local authorities (Regierung von Oberbayern, ROB-55.2-2532. Vet_02-18-143). Mice were kept under standard conditions on a 12 h light/dark cycle with access to water and food *ad libitum*. *Pde6b*^*STOP*^ mice were generated in the Barbara & Donald Jonas Stem Cells Laboratory, Columbia University, USA [16,18].

### 2.2 Immunohistochemistry

Retinal sections were incubated in primary antibodies (Table 1) in blocking solution (5 % chemiblocker #2170, MerckMillipore; and 0.3 % Triton® X-100 diluted in PBS) overnight at 4 °C. Subsequently, sections were incubated in secondary antibodies (Table 1) in PBS containing 3 % chemiblocker for 1.5 h at room temperature. For nuclear counterstaining, sections were incubated for 5 min in 5 μg/ml Hoechst 33342 (#H1399, Invitrogen).

**Table 1:** Primary and secondary antibodies.

Antibody	Host species	Dilution (IHC)	Dilution (WB)	Supplier	Catalog Number
Β-Actin-Peroxidase	Mouse	–	1:6000	Sigma–Aldrich	A3854-200UL
CD44	Rat	1:400	–	BD Pharmingen	550538
CD44	Rabbit	–	1:2000	Abcam	ab28364
GARP	Mouse	1:400	–	Sigma-Aldrich	MABN2429
GFAP	Mouse	–	1:800	Sigma–Aldrich	G3893
GLUL	Rabbit	1:2000	1:2000	Abcam	ab228590
LDHA	Rabbit	–	1:1000	Sigma–Aldrich	SAB5700695
PDE6B	Mouse	–	1:400	Santa Cruz	sc77486
PDE6B	Rabbit	1:2000	–	Thermo Fisher	PA1-722
PKM2	Rabbit	–	1:1000	Cell Signaling	#4053
pSTAT3	Mouse	–	1:2000	Cell Signaling	#4113
S100A6	Sheep	1:100	1:200	R&D Systems	AF4584
STAT3	Mouse	–	1:1000	Cell Signaling	#9139
Total OXPHOS	Mouse	–	1:400	Abcam	ab110413
488-Goat anti-Rat	Goat	1:1000	–	Thermo Fisher	A-11006
488-Donkey anti-Sheep	Donkey	1:500	–	Thermo Fisher	A-11015
488-Goat anti-Rabbit	Goat	1:1000	–	Thermo Fisher	A-11070
555-Goat anti-Mouse	Goat	1:1000	–	Thermo Fisher	A-21425
555-Goat anti-Rat	Goat	1:1000	–	Jackson	112-165-143
647-Goat anti-Rabbit	Goat	1:1000	–	Thermo Fisher	A-21245
anti-Mouse HRP	Mouse	–	1:2000	Santa Cruz	sc-516102
anti-Rabbit HRP	Mouse	–	1:2000	Santa Cruz	sc-2357
anti-Sheep HRP	Donkey	–	1:2000	Thermo Fisher	A16041

### 2.3 Quantitative analysis of ONL thickness and rod outer segment lengths

The retinal cryosections were stained with Hoechst 33342 and GARP. Images were taken in the ventral area of the retina. Using ImageJ, the ONL thickness was measured at 300 μm from the optic nerve.

### 2.4 Immunoblot

Retinas were homogenized using M-PER Mammalian Protein Extraction Reagent (Thermo #78503) containing protease inhibitor (Sigma #11697498001) and Phosphatase Inhibitor Cocktail (Cell Signaling; #5870) with a Branson Sonifier W-450D at 40 % amplitude. Proteins (20 μg per sample) were separated by SDS-PAGE and transferred to a 0.45 μm polyvinylidene difluoride (PVDF) membrane for 90 min at 90 V. Membranes were blocked in 5 % non-fat dry milk in Tris-buffered saline with Tween®20 (TBS-T) for 1 h at RT. Primary antibodies (Table 1) were incubated in 5 % non-fat dry milk overnight at 4 °C. Membranes were washed and incubated with corresponding HRP secondary antibody (Table 1) for 1 h at RT. Proteins were detected using Immobilon Forte Western HRP substrate (Millipore #WBLUF0100) and imaged using a Bio-Rad ChemiDoc MP imager.

### 2.5 ERG

ERG analysis was performed according to previously described procedures [5].

### 2.6 Proteomic profiling of whole retinal lysates and MACS-enriched retinal cell types

MACS was performed according to previously described procedures [19]. Isolated retinal cell populations and whole retinal lysates were proteolyzed with Lys-C and trypsin with filter-aided sample preparation procedure (FASP) as described [19,20]. Acidified eluted peptides were analyzed on a Q Exactive HF-X mass spectrometer (Thermo Fisher Scientific) online coupled to a UItimate 3000 RSLC nano-HPLC (Dionex). Samples were automatically injected and loaded onto the C18 trap cartridge and after 5 min eluted and separated on the C18 analytical column (Acquity UPLC M-Class HSS T3 Column, 1.8 μm, 75 μm × 250 mm; Waters) by a 90 min non-linear acetonitrile gradient at a flow rate of 250 nl/min. MS spectra were recorded at a resolution of 60000 with an AGC target of 3 x 1e6 and a maximum injection time of 30 ms from 300 to 1500 *m*/*z*. From the MS scan, the 15 most abundant peptide ions were selected for fragmentation via HCD with a normalized collision energy of 28, an isolation window of 1.6 *m*/*z*, and a dynamic exclusion of 30 s. MS/MS spectra were recorded at a resolution of 15000 with an AGC target of 1e5 and a maximum injection time of 50 ms. Unassigned charges, and charges of +1 and > 8 were excluded from precursor selection.

Acquired raw data was analyzed in the Proteome Discoverer 2.4 SP1 software (Thermo Fisher Scientific; version 2.4.1.15) for peptide and protein identification via a database search (Sequest HT search engine) against the SwissProt Human database (Release 2020_02, 20432 sequences), considering full tryptic specificity, allowing for up to one missed tryptic cleavage site, precursor mass tolerance 10 ppm, fragment mass tolerance 0.02 Da. Carbamidomethylation of cysteine was set as a static modification. Dynamic modifications included deamidation of asparagine and glutamine, oxidation of methionine, and a combination of methionine loss with acetylation on the protein N-terminus. The Percolator algorithm [21] was used for validating peptide spectrum matches and peptides. Only top-scoring identifications for each spectrum were accepted, additionally satisfying a false discovery rate <1% (high confidence). The final list of proteins satisfying the strict parsimony principle included only protein groups passing an additional protein confidence false discovery rate <5% (target/decoy concatenated search validation).

Quantification of proteins, after precursor recalibration, was based on intensity values (at RT apex) for the summed abundance of all or top3 unique peptides. Peptide abundance values were normalized on the total peptide amount. The protein abundances were calculated averaging the abundance values for admissible peptides. The final protein ratio was calculated using median abundance values of three biological replicates in a non-nested design. The statistical significance of the ratio change was ascertained by employing the approach described in [22] which is based on the presumption that we look for expression changes for proteins that are just a few in comparison to the number of total proteins being quantified. The quantification variability of the non-changing “background” proteins can be used to infer which proteins change their expression in a statistically significant manner.

### 2.7 Lactate secretion assay

The retinal lactate secretion was measured in the collected media from retinal explants. The retina was placed in DMEM low glucose (5 mM Glucose) pre-warmed to 37 °C and maintained in a cell culture incubator at 37 °C and 5% C0_2_. Media samples were collected after 15, 30 and 60 min and the concentration of lactate was measured using the Lactate Glo Luciferase Assay (#J5021, Promega) following the manufacturer's protocol. Luminometry was measured with the SpectraMax iD3 microplate reader, and the rate of lactate secretion was quantified using a standard curve.

### 2.8 Glucose consumption assay

The glucose uptake was measured similarly to the lactate secretion assay. We used the collected media obtained from retinal explants and the glucose concentration was determined using the luminometric Glucose-Glo luciferase assay kit (Promega #J6021). The rate of glucose consumption was derived by linear regression analysis.

### 2.9 Analysis of ATP

#### 2.9.1 Sample preparation

Previously published methods [23,24] were slightly modified to allow the extraction of polar metabolites from retinae. Briefly, frozen tissues were rapidly weighted into screw-capped tubes containing five extraction beads (Diagenode, Cat. No. C20000021) and resuspended in 50 μL of ice-cold water. 2.5 μL (corresponding to 512 ng) of stable isotopically labelled 15N5-ATP was added as internal standard (IS) in order to normalize signal intensities. After the addition of a mixture of chloroform/methanol/water (200 μL/250 μL/350 μL), tissues were disrupted using a Precellys homogenizer (Bertin Technologies, Montigny-le-Bretonneux, France) with 1 cycle at 6800rpm for 30sec followed by a second cycle at 5600rpm for 30sec. Samples were left 30min in ice before being centrifuged at 14,000×*g* for 15 min at 4 °C. 500 μL of the upper polar phase were collected and transferred to Amicon centrifugal filters with 3 KDa cutoff (Merck) which were previously rinsed with water according to the manufacturer's instructions. Ultrafiltration was carried out by centrifugation at 10,000×*g* for 2 h at 4 °C and filtered samples were then dried in a SpeedVac (Eppendorf, Hamburg, Germany) and stored at −70 °C. Prior to the analysis, dried samples were resuspended in 30 μL of water, vortexed, and centrifuged at 10,000×*g* for 10 min before being transferred into the corresponding nanoVial (Sciex, Concord, Ontario, Canada).

#### 2.9.2 CESI-MS analysis

Electrophoretic separation of analytes was carried out using a CESI 8000 (Sciex) equipped with a sheathless OptiMS CESI cartridge (30 μm ID x 91 cm bare fused silica capillary) maintained at 25 °C coupled to a Sciex 6600 TTOF through a NanoSpray III source. Samples were kept in a thermostated tray at 8 °C, injected hydrodynamically into the capillary at 2psi for 30sec (∼20 nL), and separated into 16 mM ammonium acetate (pH 9.7) buffer using a 30 kV voltage in normal polarity for 30min. Between injections, the capillary was rinsed with 0.1 N NaOH and 0.1 N HCl at 100psi for 2.5min each followed by water at 100psi for 3min and finally by the separation buffer at 100psi for 3min. The optimal position of the porous tip of the capillary with respect to the MS inlet was achieved by moving the XYZ stage to get a stable electrospray and the highest total ion current (TIC) signal. The values for gas 1 (GS1), gas 2 (GS2), temperature (TEM) Declustering Potential (DP), and Collision Energy (CE) were set at 0, 0, 50 °C, 40 and 8.5, respectively. MS data were acquired in positive TOF-MS mode in the *m*/*z* range of 65–900 Da using an IonSpray Voltage Spray (ISVF) of 1700 V with an accumulation time of 250 ms.

MS1 peak integration was manually performed using Skyline software [25]. ATP peak areas were normalized to their corresponding IS areas and results were expressed per mg of extracted retina.

### 2.10 Statistics

All data were plotted using GraphPad Prism 9.3. As indicated in figure legends, quantitative data are presented as mean ± SEM. All data consisting of two groups (e.g., lactate, ATP) were analyzed with the unpaired t-test. For multiple comparisons, differences were analyzed using one-way ANOVA followed by Tukey's multiple comparisons post hoc test. MACS data (Figure 6) were analyzed using two-way ANOVA followed by Bonferroni post hoc test. The minimum level of significance was defined as *P* < 0.05 and is indicated by the p-value (∗*P* ≤ .05; ∗∗*P* ≤ .01; ∗∗∗*P* ≤ .001). The N values refer to the number of individual animals for the respective genotype. Proteomic data analyses were performed using R (version 4.3.2) and package ‘limma’ (version 3.58) for differential expression.

## 3 Results

### 3.1 *Pde6b* gene restoration rescued PDE6 subunits

**Figure 1:**
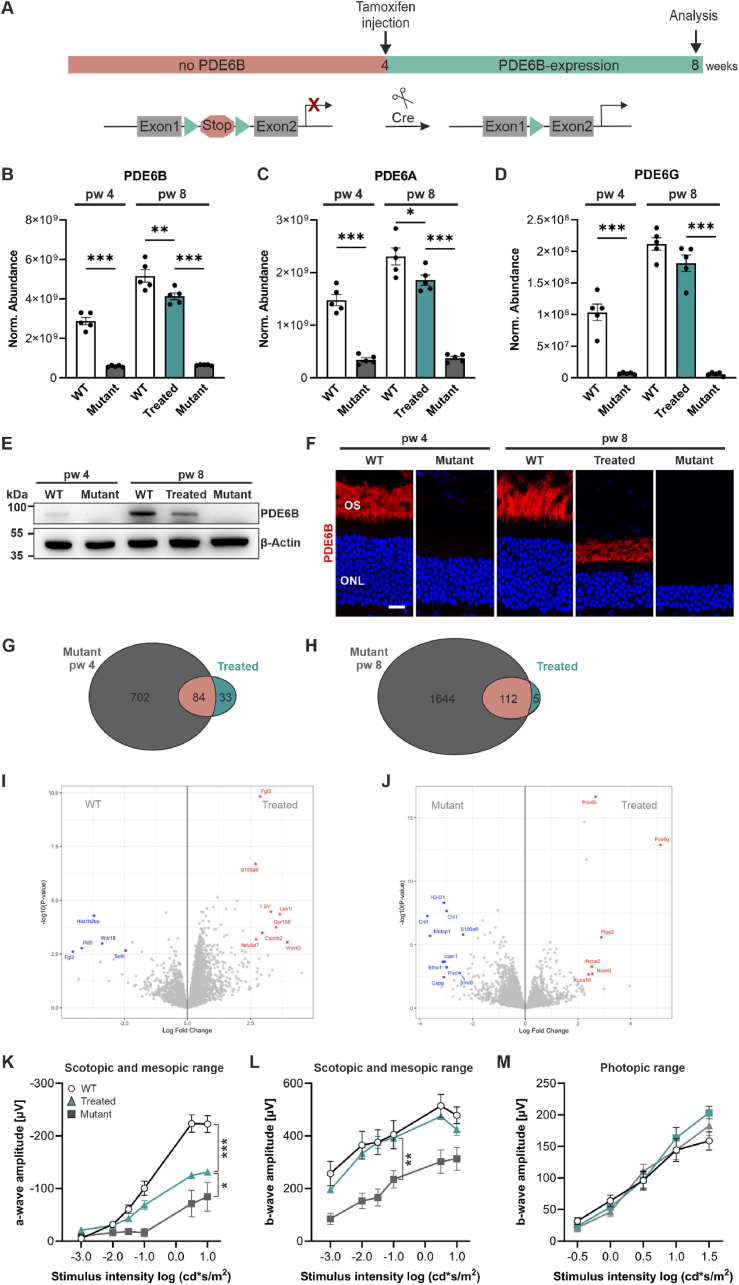
***Pde6b* restoration rescued PDE6 expression and most but not all dysregulated proteins. (A)** Schematic representation of experiment. Tamoxifen injection (at 4 weeks) activates CreERT2 recombinase, which splices out the stop cassette, leading to PDE6B expression. **(B**–**J)** WT, mutant, and treated retinas were analyzed at 4 and/or 8 weeks of age. Treated mice were tamoxifen-injected at 4 weeks of age. **(B-D, G-J)** Retinas were analyzed by label-free liquid chromatography-tandem mass spectrometry (LC-MS/MS)-based proteomics. **(B**–**D)** Expression of PDE6 subunits was reduced in mutant and restored in treated mice. **(E)** Representative PDE6B immunoblot of retinal lysates. β-Actin was used as a loading control. **(F)** Representative images of retinal sections immunostained for PDE6B and counterstained with Hoechst 33342. Scale bar, 15 μm. **(G, H)** Venn diagrams representing the number of unique or overlapping proteins that were differentially expressed in 4-week-old-mutant and treated mice (FDR<0.1) **(G)** and in 8-week-old mutant and treated mice **(H)** in comparison to 8-week-old WT mice. **(I, J)** Volcano plots showing differentially expressed proteins between 8-week-old WT and treated **(I)** and 8-week-old mutant and treated **(J)** retinas. Proteins with fold change >5, and FDR<0.1 are highlighted. **(K)** Scotopic (−3 and −2 log cd × s/m^2^) and mesopic a-wave amplitudes. **(L)** Scotopic (−3 and −2 log cd × s/m^2^) and mesopic b-wave amplitudes. **(M)** Photopic b-wave amplitudes. **(**K–M**)** N = 5 for WT and treated, N = 7 for mutant. **(B-D, K-M)** Data, presented as mean ± SEM, were compared by ANOVA. ∗*P* ≤ 0.05; ∗∗*P* ≤ 0.01; ∗∗∗*P* ≤ 0.001.

In this study, we used the RP gene therapy mouse model *Pde6b*^*STOP/STOP*^*, Pde6g*^*CreERT2/*+^ to understand alterations in the proteome in response to photoreceptor degeneration, and how Cre-mediated *Pde6b* gene restoration impacts these changes. In *Pde6b*^*STOP/STOP*^*, Pde6g*^*CreERT2/*+^ mice, the floxed stop cassette in both *Pde6b* alleles prevents PDE6B expression, leading to photoreceptor degeneration [5]. Photoreceptor loss can be quantified by measuring the thickness of the outer nuclear layer (ONL). The ONL thickness in *Pde6b*^*STOP/STOP*^*, Pde6g*^*CreERT2/*+^ mice had decreased by approximately 34 % and 60% at 4 and 8 weeks of age, respectively (compared with age-matched WT) (Supplementary Figs. S1A, B). The rod outer segment length was reduced by about 50% and 70% at 4 and 8 weeks of age, respectively (compared with age-matched WT) (Supplementary Figs. S1A, C). We injected these mice with tamoxifen at 4 weeks and analyzed the retinal proteome at 8 weeks of age (Figure 1A). After tamoxifen injection, the Cre recombinase is activated, excising the stop cassette, leading to PDE6B expression which halts photoreceptor degeneration and rod outer segment shortening (Supplementary Figs. S1A-C). Retinas from 5 groups were subjected to label-free liquid chromatography-tandem mass spectrometry (LC-MS/MS) based proteomics: untreated *Pde6b*^*STOP/WT*^*, Pde6g*^*CreERT2/*+^ (referred to as WT) and *Pde6b*^*STOP/STOP*^*, Pde6g*^*CreERT2/*+^ (referred to as mutant) mice at 4 weeks of age, and treated WT, treated mutant (referred to as treated) and untreated mutant mice at 8 weeks of age (Supplementary Figs. S1D-G). The treated WT were also tamoxifen injected at 4 weeks of age to account for the effects of tamoxifen.

To assess the quality of the proteomic data, we quantitatively compared the abundance of the three phosphodiesterase 6 (PDE6) subunits: beta (Figure 1B), alpha (Figure 1C), and gamma (Figure 1D). PDE6B expression was highest in 8-week-old treated WT mice (2 functional *Pde6b* alleles) and decreased by about half in 4-week-old untreated heterozygous WT mice (1 functional *Pde6b* allele). As expected, in mutant mice (at both 4- and 8 weeks of age), PDE6B expression was significantly reduced. PDE6B expression was restored in treated mice (2 functional *Pde6b* alleles) (Figure 1B) and it was only slightly reduced compared to 8-week-old WT. Given that the ONL thickness in treated mice is reduced (Supplementary Fig. S1B), these data suggest higher PDE6B abundance per rod than in WT. We observed similar expression patterns across the different groups for PDE6A (Figure 1C) and PDE6G (Figure 1D). PDE6A and PDE6G expression was also significantly reduced in 4- and 8-week-old mutant mice (Figure 1C,D); this was expected, since loss of PDE6B-subunit prevents the formation of the heterotetrameric PDE6 complex and leads to the degradation of the remaining subunits [26,27]. Thus, our quintuplicate proteome analysis confirms the reduction and restoration of PDE6 subunits in mutant and treated retinas, respectively. The PDE6B expression was validated by immunoblots, where we detected PDE6B only in WT and treated retinas – with the highest expression in 8-week-old WT retinas (Figure 1E). It was also validated by immunohistochemistry (IHC), where we detected PDE6B in photoreceptor outer segments in WT and treated retinas, but not in mutant retinas (Figure 1F).

Next, we compared the number of unique and overlapping proteins that were differentially expressed in 4-week-old mutant and 8-week-old treated mice in comparison to 8-week-old WT mice (FDR<0.1) (Figure 1G). Out of the total 4104 proteins identified, 702 and 33 proteins were exclusively expressed in mutant and treated mice, respectively. Additionally, 84 proteins were significantly different in both mutant and treated mice compared to WT (Figure 1G). We further examined unique and overlapping proteins that were differentially expressed in 8-week-old mutant and treated mice in comparison to 8-week-old WT (FDR<0.1) (Figure 1H). 1644 and 5 were exclusively expressed in 8-week-old mutant and treated mice, respectively. 112 proteins were differentially expressed in both treated and mutant mice. These analyses show that 19% and 43% of proteins were dysregulated in mutant mice at 4 weeks and 8 weeks of age, respectively. Only 3% of proteins were dysregulated in treated mice compared to WT, demonstrating that most dysregulated proteins were restored and that treatment prevented most of the changes in the proteome.

Volcano plots were used to visualize proteins with differences in expression (highlighted proteins, fold change >5, FDR<0.1) between 8-week-old WT and treated mice (Figure 1I) as well as between 8-week-old mutant and treated mice (Figure 1J). In summary, these proteomic data provide a comprehensive resource on the dynamics occurring in the proteome of treated and untreated RP retinas.

To evaluate the success of *Pde6b* restoration on retinal function, full-field single-flash electroretinography (ERG) responses were recorded in WT, treated, and mutant mice. For WT animals, we utilized the ERG data previously published [5] (Figure 1K-M). In mutant mice, the a-wave (negative deflection), generated by photoreceptor cells, was significantly smaller compared to treated mice at light intensities of - 1.0, 0.5, and 1.0 log(cd × s/m^2^) (P ≤ 0.05). In treated mice, the scotopic a-wave response was improved (Figure 1K). The b-wave amplitude (positive deflection), generated by bipolar cells, was fully restored to WT levels (Figure 1L). After light-adaption, to derive cone-response, the b-wave amplitude was measured. There was no significant difference between the groups, showing that cone photoreceptor function remained unaffected at this disease stage (Figure 1M).

### 3.2 *Pde6b* gene restoration increased expression of phototransduction proteins

**Figure 2:**
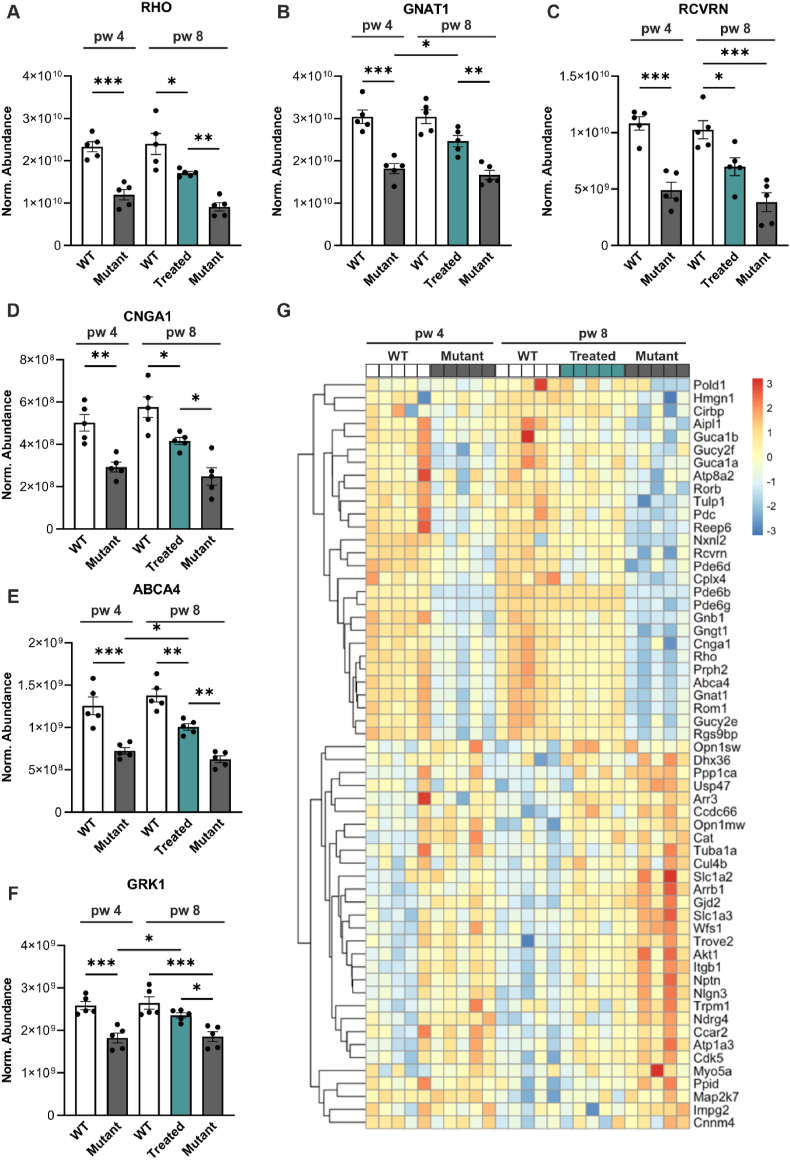
***Pde6b* gene restoration increased expression of proteins involved in phototransduction.** Retinas from WT, mutant, and treated mice were analyzed at 4 and/or 8 weeks of age by label-free liquid chromatography-tandem mass spectrometry (LC-MS/MS)-based proteomics. Treated mice were tamoxifen-injected at 4 weeks of age. **(A**–**F)** RHO **(A),** GNAT1 **(B),** RCVRN **(C),** CNGA1 **(D),** ABCA4 **(E),** and GRK1 **(F)** are essential for the phototransduction cascade and were significantly downregulated in mutant compared to WT retinas. Their expression could be restored in treated mice. **(G)** Heat map representation of proteins involved in sensory perception of light stimulus. **(A**–**F)** Data, presented as mean ± SEM, were compared by ANOVA. ∗*P* ≤ 0.05; ∗∗*P* ≤ 0.01; ∗∗∗*P* ≤ 0.001.

In RP, rod degeneration initially manifests as shortening of outer segments [28,29]. Given that the phototransduction cascade occurs in the outer segment of photoreceptors [30], we investigated the expression of key proteins for this cascade (Figure 2). Rhodopsin (RHO), G protein subunit alpha transducin 1 (GNAT1), recoverin (RCVRN), cyclic nucleotide-gated channel subunit alpha 1 (CNGA1), ATP binding cassette subfamily A member 4 (ABCA4), and G protein-dependent receptor kinase 1 (GRK1) were highly expressed in both 4- and 8-week-old WT mice (Figure 2A–F). In mutant mice, these proteins were reduced, indicating diminished rod outer segment lengths and phototransduction potential. The expression levels were not changed between 4- and 8-week-old mutant mice, despite the more advanced degeneration at 8 weeks. Restoration of PDE6B led to a significant upregulation of all these proteins in treated retinas compared to 8-week-old mutants. GNAT1 (Figure 2B), ABCA4 (Figure 2E), and GRK1 (Figure 2F) were significantly upregulated in treated retinas compared to 4-week-old mutants (the timepoint of treatment). The restoration of phototransduction proteins indicates high plasticity of the rod photoreceptors following successful gene therapy.

To gain a more comprehensive understanding of protein expression related to phototransduction, we visualized the expression of proteins involved in the sensory perception of light stimulus (gene ontology (GO) terms 50953, 9583, 9416, 7602, 50962) (Figure 2G). The first 28 displayed proteins, ranging from POLD1 to RGS9BP, were downregulated in mutant mice at both 4 and 8 weeks of age, whereas the proteins in the treated group exhibited expression levels similar to those in WT animals, underscoring the robust plastic capacity following *Pde6b* gene restoration. Conversely, several proteins, such as Arrb1, Gjd2, Nlgn3, and Trpm1, play important roles in G-protein receptor coupling and thereby regulating the signal-to-noise-ratio, were upregulated in 8-week-old mutant mice, which was halted by treatment [[31], [32], [33], [34]].

### 3.3 *Pde6b* gene restoration halted gliotic müller cell response

**Figure 3:**
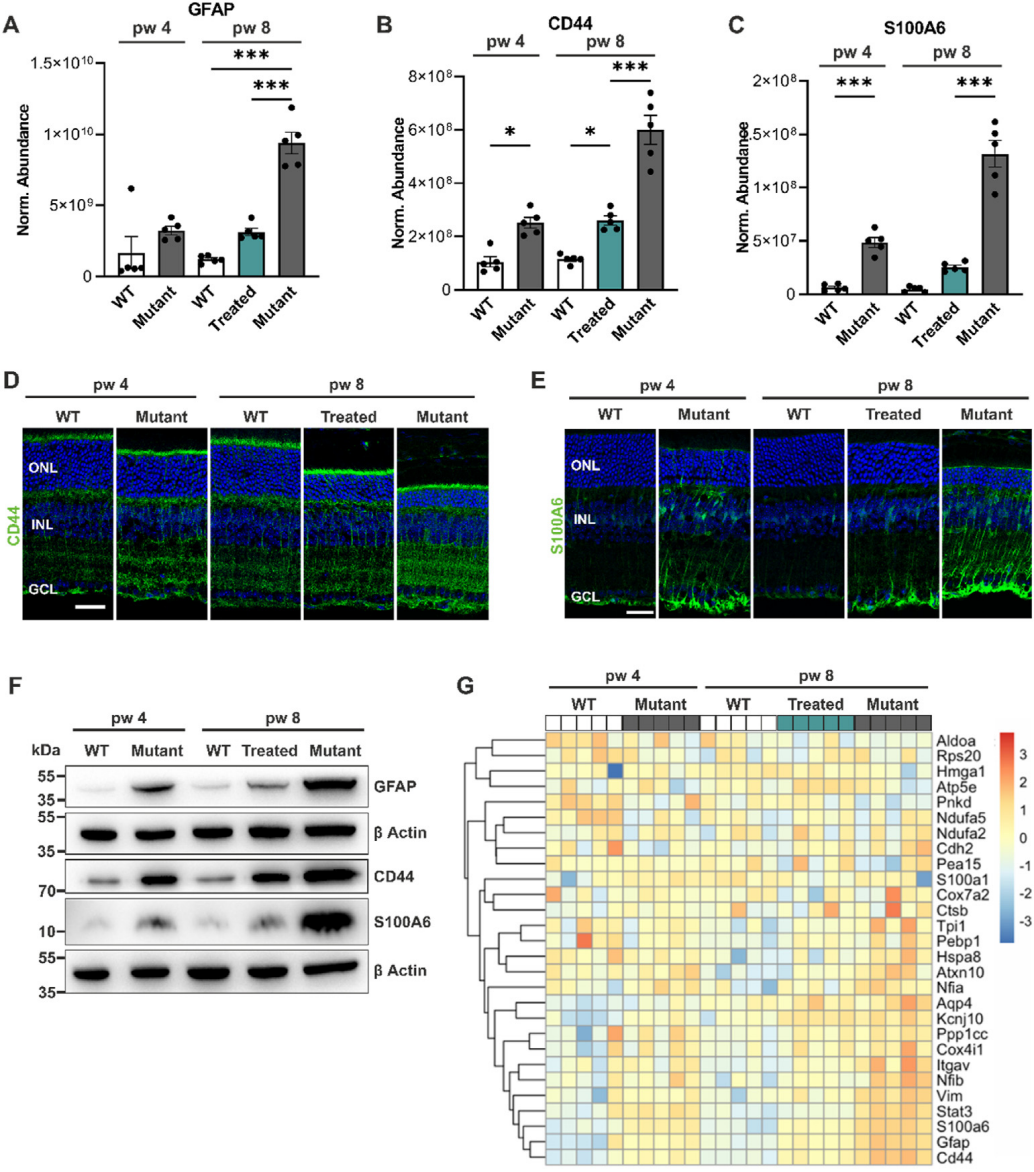
***Pde6b* gene restoration halted activation of Müller cells.** WT, mutant, and treated retinas were analyzed at 4 and/or 8 weeks of age. Treated mice were tamoxifen-injected at 4 weeks of age. **(A-C, G)** Retinas were analyzed by label-free liquid chromatography-tandem mass spectrometry (LC-MS/MS)-based proteomics **(A)** Quantitative analysis of the gliosis marker glial fibrillary acidic protein (GFAP) expression revealed significantly higher levels in mutant compared to treated and WT retinas at 8 weeks of age. **(B, C)** The quantitative analysis of CD44 and S100A6 revealed significantly higher levels of CD44 **(B)** and S100A6 **(C)** in mutant compared to treated mice at 8 weeks of age. **(A**–**C)** Data, presented as mean ± SEM, were compared by ANOVA. ∗*P* ≤ 0.05; ∗∗∗*P ≤* 0.001. **(D, E)** Representative images of retinal sections immunostained for CD44 **(D)** and S100A6 **(E).** Both proteins are exclusively expressed in Müller cells. Scale bar, 15 μm. **(F)** Representative GFAP, CD44, and S100A6 immunoblots of retinal lysates. β-Actin was used as a loading control**. (G)** Heat map representation of gliosis-associated proteins. ONL, outer nuclear layer; INL, inner nuclear layer; GCL, ganglion cell layer.

Müller cells play a crucial role in maintaining retinal homeostasis and support the structure and function of photoreceptor cells [6,35]. In response to retinal degeneration, Müller cells undergo gliosis, a process marked by upregulation of several proteins. To investigate the Müller cell response following treatment, we examined key proteins for Müller cell activation (Figure 3). Glial fibrillary acidic protein (GFAP) (Figure 3A), CD44 (Figure 3B) and S100A6 (Figure 3C) expression was low in both 4- and 8-week-old WT mice. CD44 and S100A6 expression increased in 4-week-old mutant mice (vs age-matched WT), and all 3 proteins were further increased in 8-week-old mutant mice (vs age-matched WT; P ≤ 0.0001). GFAP and CD44 expression levels were similar in 4-week-old mutant and 8-week-old treated retinas, suggesting that treatment did not yet reverse the increased expression to WT levels. S100A6 expression, however, was almost restored to WT-level (Figure 3C). These data were validated by IHC, which demonstrated the expression of CD44 predominantly in the apical microvilli of Müller cells (Figure 3D) and S100A6 expression in the endfeet of Müller cells from both treated and mutant retinas (Figure 3E). Additionally, we detected highest expression of GFAP, CD44, and S100A6 in 8-week-old mutant retinas by immunoblot (Figure 3F). For a comprehensive understanding of the Müller cell response following *Pde6b* gene restoration, we generated a heatmap displaying gliosis-associated proteins identified in several publications addressing Müller cell response post-injury [6,[36], [37], [38]] (Figure 3G). In 4- and 8-week-old WT mice, the expression of most of these proteins is minimal, increased levels are observed in 4- and 8-week-old mutant and treated mice. Based on these observations, we conclude that rescue of PDE6B halts Müller cell gliosis; however, not all gliosis-associated proteins were fully restored to WT levels 4 weeks post-treatment.

### 3.4 *Pde6b* gene restoration largely inactivates pro-inflammatory proteins

**Figure 4:**
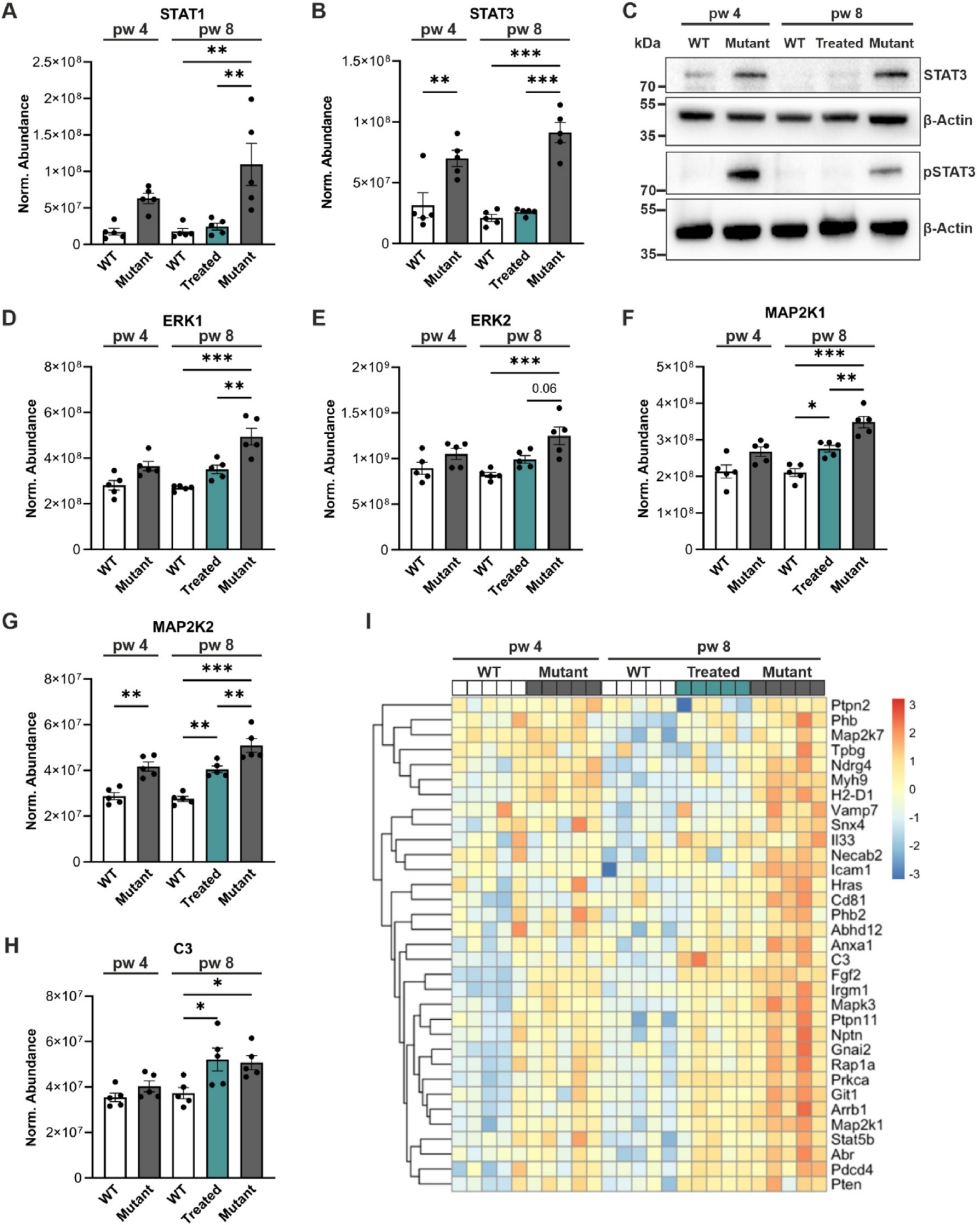
***Pde6b* gene restoration reversed/halted the activation of the JAK-STAT and MAPK pathways.** WT, mutant, and treated retinas were analyzed at 4 and/or 8 weeks of age. Treated mice were tamoxifen-injected at 4 weeks of age. **(A-B, D-I)** Retinas were analyzed by label-free liquid chromatography-tandem mass spectrometry (LC-MS/MS)-based proteomics**. (A**–**B)** Quantitative analysis of STAT1 **(A)** and STAT3 **(B)**. **(C)** Representative STAT3 and pSTAT3 immunoblot of retinal lysates. β-Actin was used as a loading control. **(D–E)** Quantitative analysis of ERK1 **(D)**, and ERK2 **(E). (F–G)** Quantitative analysis of MAP2K1 **(F)** and MAP2K2 **(G). (H)** Quantitative analysis of complement component 3 (C3) expression. **(I)** Heat map representation of proteins involved in positive regulation of ERK1/2 cascade (GO 0070374, GO 0050727). **(A-B, D-H)** Data, presented as mean ± SEM, were compared by ANOVA. ∗*P* ≤ 0.05; ∗∗*P* ≤ 0.01; ∗∗∗*P* ≤ 0.001.

In RP, the initial mutation-driven photoreceptor degeneration leads to chronic inflammation, marked by the activation of the Janus kinase-signal transducer and activator transcription (JAK-STAT) and the mitogen-activated protein kinase (MAPK) pathway [4,13,39]. To determine whether our *Pde6b* gene restoration approach could halt or reverse these inflammatory pathways, we analyzed different key inflammatory factors (Figure 4). Our proteomic analysis revealed significantly higher levels of STAT1 in 8-week-old mutant mice (vs age-matched WT; P = 0.001). Notably, this increase was prevented in the treated retina (Figure 4A). STAT3 was significantly upregulated in both 4- and 8-week-old mutant mice (vs age-matched WT; P < 0.001). In WT and treated mice, STAT3 protein levels were similar, suggesting that treatment reversed the increased STAT3 expression (Figure 4B). The decreased STAT3 levels in treated retinas were validated by immunoblot (Figure 4C). Since STAT3 is activated by phosphorylation, we next analyzed phosphorylated STAT3 expression by immunoblot. Phosphorylated STAT3 was not detected in WT and treated retinas, but increased in mutants, indicating that PDE6B rescue also reversed STAT3 signaling activation (Figure 4C). Activation of the MAPK pathway primarily involves extracellular signal-regulated kinases 1/2 (ERK 1/2) [40,41]. ERK1 and ERK2 were significantly upregulated in mutant mice at 8 weeks of age compared to age-matched WT controls, which was prevented in treated mice (Figure 4D,E). Important upstream regulators of ERK1/2 are MAP2K1 and MAP2K2 [42,43], which were both significantly upregulated in mutant and treated retinas at 8 weeks of age (Figure 4F,G).

Another crucial pathway of the innate immune response is the complement system. Studies have demonstrated that complement component C3 plays an important role in microglia–photoreceptor interaction [[44], [45], [46]]. Our proteomic data revealed significantly higher C3 levels in both treated and mutant mice at 8 weeks of age compared to age-matched WT controls (Figure 4H). We next visualized proteins correlated with the upregulation of the ERK1/2 cascade (GO term 0070374, GO 0050727) (Figure 4I). All proteins were highly expressed in mutant mice at 8 weeks of age, while the expression was similar in treated mice and 4-week-old mutant mice. These data show that *Pde6b* gene restoration reversed and halted the initiation/activation of the STAT pathway and ERK pathway, respectively. Further investigation is needed to determine whether additional time is required for the ERK pathway to return to WT levels after rescue.

### 3.5 *Pde6b* gene restoration did not decrease expression of OXPHOS-related proteins to WT

**Figure 5:**
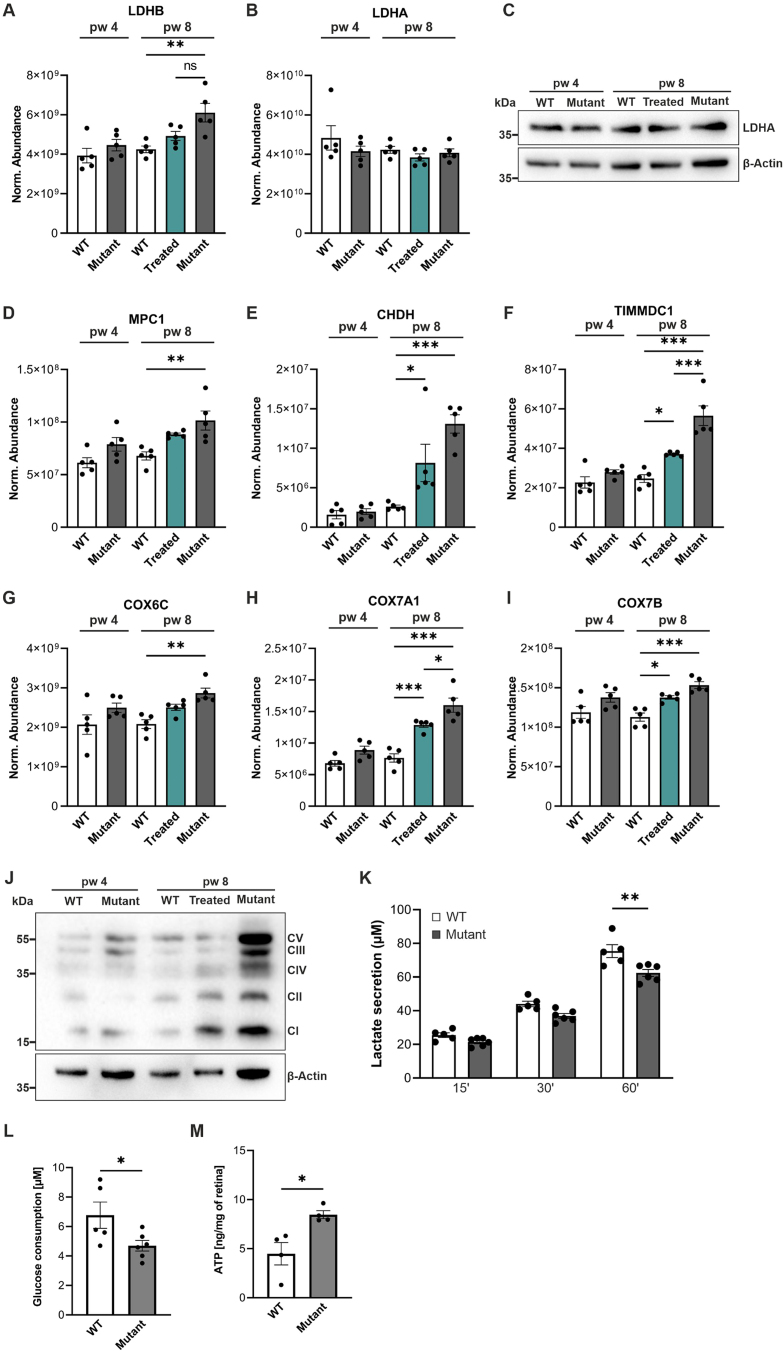
**Upregulation of OXPHOS related proteins in treated and mutant retinas. (A, B, D–I)** WT, mutant, and treated retinas were analyzed at 4 and/or 8 weeks of age. Treated mice were tamoxifen-injected at 4 weeks of age. Retinas were analyzed by label-free liquid chromatography-tandem mass spectrometry (LC-MS/MS)-based proteomics. **(A)** Quantitative analysis of LDHB. **(B)** Quantitative analysis of LDHA expression. **(C)** Representative LDHA immunoblot of retinal lysates. β-Actin was used as a loading control. **(D**–**I)** Quantitative analysis of the OXPHOS proteins MPC1 **(D)**, CHDH **(E)**, TIMMDC1 **(F),** and cytochrome C oxidase (COX) nuclear-encoded subunits COX6C **(G)**, COX7A1 **(H)**, COX7B **(I)**. **(A, B, D–I)** Data, presented as mean ± SEM, were compared by ANOVA. ∗*P* ≤ 0.05; ∗∗*P* ≤ 0.01; ∗∗∗*P* ≤ 0.001. **(J)** Representative immunoblot of the OXPHOS complexes I–V of retinal lysates. β-Actin was used as a loading control. **(K)** Lactate secretion from retinal explants after 15, 30, and 60 min from WT (week 10) and mutant (week 8) mice. **(L)** Glucose consumption from retinal explants after 60 min from WT (week 10) and mutant (week 8) mice. **(M)** ATP analysis showed increased levels in mutant retinas compared to WT at 8 weeks of age. **(L**–**M)** Data, presented as mean ± SEM, were compared by unpaired t-test. ∗*P* ≤ 0.05.

Photoreceptors exhibit a high energy demand and are among the most metabolically active cells in the body, maintaining phototransduction, neurotransmission, and constant outer segment morphogenesis [[47], [48], [49]]. It has been suggested that photoreceptors predominantly rely on aerobic glycolysis in order to continuously renew their outer segment disks [50,51]. To investigate metabolic changes in our mutant and treated mice, we first examined protein levels of glycolysis markers. For example, lactate dehydrogenase (LDH) isoforms plays a pivotal role in aerobic glycolysis and convert pyruvate to lactate and vice versa [52]. LDHB, responsible for converting lactate to pyruvate, was significantly upregulated in 8-week-old mutant mice compared to age-matched WT controls. It was also slightly upregulated in treated mice compared to WT (P = 0.2) (Figure 5A). On the other hand, LDHA, responsible for converting pyruvate to lactate and therefore associated with aerobic glycolysis [49], exhibited similar expression levels across all 5 mouse groups (Figure 5B), which was confirmed by LDHA immunoblotting (Figure 5C). Another critical enzyme in glycolysis is pyruvate kinase, which catalyzes the final step of this metabolic pathway. The dimer form, pyruvate kinase M2 (PKM2), regulates the rate-limiting step of glycolysis, thereby directing glucose metabolism to lactate production [53]. Since PKM2 was not detected in our proteomics data, we performed immunoblot (Supplementary Figs. S2A and B) and qRT-PCR analysis (Supplementary Fig. S2C). PKM2 appeared to be downregulated in treated and mutant retinas compared to age-matched WT controls, but these differences are not statistically significant (Supplementary Fig. S2). To understand changes in theoxidative phosphorylation (OXPHOS) pathway, we next analyzed respiratory chain markers. For example, mitochondrial pyruvate carrier 1 (MPC1), which shuttles pyruvate into the mitochondrial matrix, was significantly upregulated in mutant retinas at 8 weeks of age (compared to WT) (Figure 5D). Choline dehydrogenase (CHDH), a key mitochondrial enzyme, and translocase of inner mitochondrial membrane domain containing 1 (TIMMDC1), which is involved in the assembly of mitochondrial complex I, were upregulated in both 8-week-old treated and mutant retinas compared to age-matched WT-controls (Figure 5E,F). Moreover, we analyzed subunits of cytochrome C oxidase (COX), the terminal enzyme in the mitochondrial electron transport chain [54,55]. COX6C (Figure 5G), COX7A1 (Figure 5H), and COX7B (Figure 5I) were significantly upregulated in 8-week-old mutant retinas compared to age-matched WT controls. COX7A1 and COX7B were also significantly upregulated in treated mice. To validate the upregulation of the enzymes of the respiratory chain, we performed immunoblotting using an antibody cocktail capable of detecting the OXPHOS complexes I–V. Notably, all complexes were highly expressed in 8-week-old mutant retinas. Furthermore, complexes I, II, and III were upregulated in treated mice compared to age-matched WT controls (Figure 5J). Since these data suggest a decreased aerobic glycolysis and an increased OXPHOS rate in mutant retinas, we next analyzed lactate secretion, glucose consumption and ATP levels using enzymatic lactate/glucose assays and capillary electrophoresis coupled to mass spectrometry (CESI-MS), respectively. We observed a significantly reduced lactate secretion (Figure 5K) and glucose consumption (Figure 5L) after 60 minutes in retinal explants from 8-week-old mutant retinas compared to 10-week-old WT and significantly higher ATP levels in mutant retinas (P ≤ 0.05) (Figure 5M).

**Figure 6:**
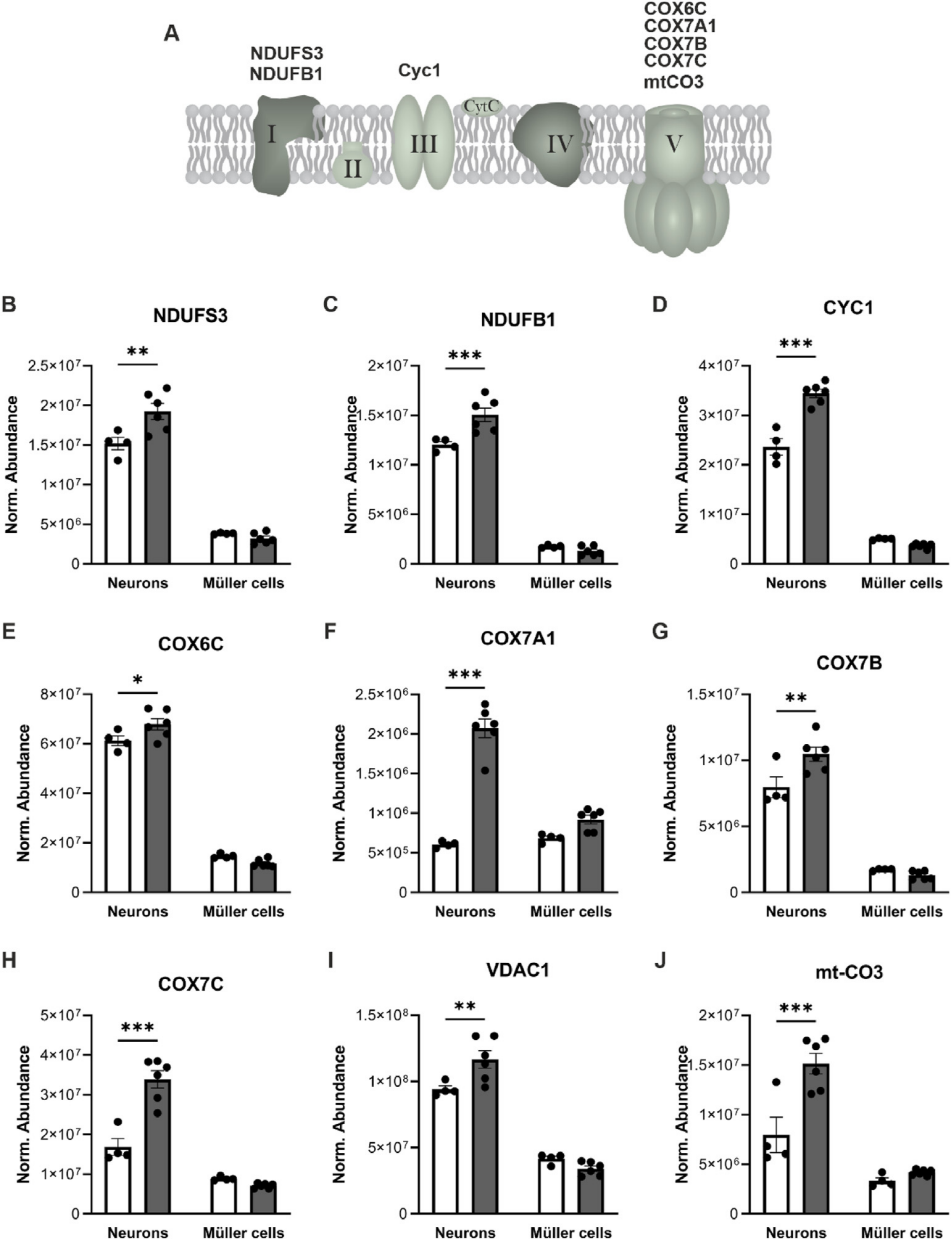
**Upregulation of OXPHOS-related proteins in neurons from mutant retinas.(A)** Simplified scheme of the electron transport chain located within the inner mitochondrial membrane. **(B**–**J)** MACS enriched Müller cell and neuronal cell fractions from 8-week-old WT and mutant retinas were subjected to label-free liquid chromatography-tandem mass spectrometry (LC-MS/MS)-based proteomics. Quantitative analysis of NDUFS3 **(B)**, NDUFB1 **(C)**, CYC1 **(D)**, COX6C **(E)**, COX7A1 **(F)**, COX7B (**G)**, COX7C **(H),** VDAC1 **(I)** and mt-CO3 **(J)**. **(B**–**J)** Data, presented as mean ± SEM, were compared by two-way ANOVA followed by Bonferroni post hoc test. ∗*P* ≤ 0.05; ∗∗*P* ≤ 0.01; ∗∗∗*P* ≤ 0.001.

In the retina, photoreceptors and Müller cells undertake the metabolic burden of glucose metabolism. To understand whether the upregulation of these OXPHOS markers (Figure 5) is cell-specific, we performed proteomic analysis on Müller cells and neurons isolated from 8-week-old WT and mutant retinas using a multistep magnetic-activated cell sorting (MACS) procedure. We consistently observed a significant increase in proteins of complex I (NDUFS3 (Figure 6B) and NDUFB1 (Figure 6C)), complex III (CYC1 (Figure 6D)), and complex V (COX6C (Figure 6E), COX7A1 (Figure 6F), COX7B (Figure 6G) and COX7C (Figure 6H)) of the electron transport chain (Figure 6A) in the neuronal cell fraction from mutant mice compared to WT. Additionally, the voltage-dependent anion channel 1 (VDAC1) (Figure 6I), a key protein regulating mitochondrial function [56], and mitochondrially encoded cytochrome C oxidase III (mt-CO3) (Figure 6J), which reflects the mitochondrial metabolic status [57] were also significantly upregulated in neurons from mutant mice compared to WT. These findings suggest that neurons, rather than Müller cells, increase their OXPHOS rate.

Collectively, these data suggest that photoreceptor degeneration leads to an upregulation of proteins involved in mitochondrial OXPHOS.

## 4 Discussion

**Figure 7:**
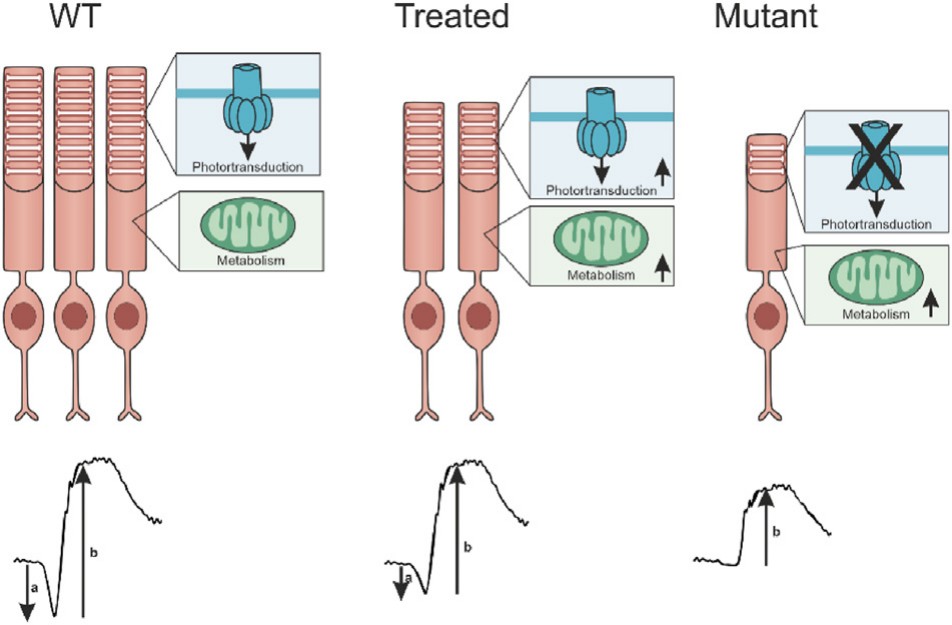
Graphical summary.

While gene therapy offers promising prospects for curing RP, recent clinical trial data on retinal degenerative diseases have raised sustainability concerns [9,11,58]. It becomes urgent to understand how therapeutical interventions modulate the molecular and cellular changes experienced by the retina. In this study, we performed an untargeted proteomic analysis on retinas from a RP gene therapy mouse model to determine which aspects could be restored by treatment. Our results may be gene-specific and may not apply to all types of RP models – given their diverse etiologies. We showed that genetic restoration of the *Pde6b*-gene can halt photoreceptor degeneration and restore visual function. Proteins involved in the phototransduction cascade were upregulated post-treatment, and both gliotic Müller cell and retinal pro-inflammatory response could be halted or restored to WT levels. We observed an increase in proteins related to the OXPHOS pathway in the degenerative retina, which could not be restored by treatment. These findings are summarized in (Figure 7).

Mutations in rod phototransduction proteins cause rod outer segment shortening and photoreceptor degeneration. We also observed progressive shortening of rod outer segments and a reduction in photoreceptor number in our *Pde6b*^*STOP/STOP*^ RP mouse model (Supplementary Fig. 1B,C). The rod outer segments are filled with a dense stack of membrane discs, which contain the proteins of the phototransduction cascade. We analyzed the expression levels of some phototransduction proteins and found, on the one hand, that the decreased rod outer segment length and/or the decreased number of rods in the mutant mice was reflected by a decreased expression of rod phototransduction proteins (Figure 2). On the other hand, the expression level of rod phototransduction proteins was similar in 4- and 8-week-old mutant mice, even though the degeneration was more advanced at 8 weeks. Thus, the length of the rod outer segments and/or the number of rods correlate partially with the expression of the phototransduction proteins, but there might also be some compensatory mechanisms. In addition, our data show that rod phototransduction proteins (including PDE6) were upregulated following tamoxifen-mediated *Pde6b*-gene restoration (Figure 1, Figure 2), even though shortened outer segments do not regrow to their normal length (Supplementary Fig. S1C). This upregulation suggests that there are more PDE6 copies per rod than in WT, which could functionally compensate for the decreased rod outer segment lengths and rod numbers. Our ERG data demonstrate that the a-wave amplitude (reflecting the response of photoreceptors [59]) is reduced in treated mice compared to WT (Figure 1K), since about 34% of photoreceptors have degenerated. Moreover, the b-wave amplitude (reflecting downstream retinal neurons, including bipolar cells) was fully restored (Figure 1L), indicating that the input loss from the photoreceptors was compensated, which could be partially accounted for the increased abundance of phototransduction proteins in remaining photoreceptors.

Retinal degeneration triggers the activation of Müller cells, a process known as gliosis [60]. Müller cells, serving as the main support cells of the healthy retina [61], exhibit increased GFAP synthesis under gliotic conditions [62]. In addition, gliotic Müller cells form a dense fibrotic layer in the subretinal space, which isolates the neural retina from the RPE [63]. This glial seal could impede further gene therapy [64]. While a transient inflammation can serve as a helpful response to insults such as tissue damage, a chronic response that triggers the secretion of pro-inflammatory cytokines can be pathogenic. Therefore, it's important to investigate the Müller cell response following RP gene therapy. Our findings showed that the expression of proteins associated with Müller cell gliosis could be halted but not fully restored to WT levels post-treatment. This may be attributed to the relatively short time frame of 4 weeks between treatment and proteome analysis, suggesting that complete restoration of these proteins to WT levels would require more time. Since Müller cell activation can also regulate inflammatory responses [65] and induce microglia activation [13,66], we investigated how different inflammatory pathways respond to our idealized gene therapy scenario. We have previously demonstrated that an increased number of activated microglia accompanies photoreceptor degeneration in our *Pde6b*^*STOP/STOP*^ mouse model, which was reversed by *Pde6b* gene restoration [67].

In RP, both microglia and Müller cells activate the MAPK/ERK and JAK/STAT pathways, which leads to the release of pro-inflammatory cytokines like TNFα [4,65]. It has been reported that p-ERK immunosignal was located in GFAP-positive Müller cells after LPS-induced inflammation [68], and co-localized with the Müller cell-specific marker glutamine synthetase in retinal explants in response to GDNF stimulation [69]. STAT3 was found in some ganglion cells and Müller cells [70,71]. Both of these pathways were upregulated in 8-week-old mutant mice compared to age-matched WT controls, and while JAK/STAT response was restored in treated mice, MAPK/ERK response persisted, suggesting that these two pathways play different roles in regulating retinal inflammatory responses. These findings show that *Pde6b* gene restoration inactivates certain pro-inflammatory pathways. The elevated levels of cytokines likely require more than 4 weeks to decrease, but have no detrimental effects on photoreceptors, as the ONL thickness of treated mice was similar to the ONL thickness of 4-week-old mutant mice. Examination of inflammatory markers and gliosis activation at further time points would be required to test this possibility. Otherwise, if the glial inflammatory response does not resolve, this warrants further investigation as this could compromise the success of gene therapy and needs to be considered in therapy development, for instance by controlling the inflammatory response and employment of neuroprotective approaches.

We observed elevated expression of complement component 3 (C3) in treated mice compared to WT. The complement system is part of the innate immune system, with C3 being an essential component of complement activation [44,72]. Previous studies have shown that C3-mediated complement activation is essential for maintaining normal retinal function and mediating the essential clearance of apoptotic photoreceptors by microglia [44,45]. The increase in C3 levels post-treatment is probably an immunomodulatory response strategy of the remaining photoreceptors. Further research is required to comprehensively understand complement activation post-treatment, particularly investigating the expression of other complement components and regulatory factors.

It has been suggested that photoreceptors predominantly rely on aerobic glycolysis in order to continuously renew their outer segment disks [50,51]. In aerobic glycolysis, most glucose is converted to lactate rather than catabolizing it completely to carbon dioxide via OXPHOS to generate ATP. Glucose is transported from the choroidal blood to the photoreceptors via the RPE. Several RP animal models predict glucose shortage in photoreceptors [[73], [74], [75]]. It has been proposed that in RP the RPE holds on to glucose, which leads to decreased transfer of glucose to photoreceptors, starvation and subsequent shortening of the outer segments and degeneration [75]. Our data revealed significant upregulation of mitochondrial markers and increased ATP production in the mutant retina. While both photoreceptors and Müller cells are metabolically active, this effect accounts in particular to the neurons. These data indicate that the metabolic demands of photoreceptors differ in RP. These metabolic changes were not restored in our treated retinas. Additionally, lactate secretion was diminished in mutant retinas, suggesting a decreased aerobic glycolysis, but further experiments are required to test this hypothesis. The increase in OXPHOS-related proteins coupled with the decrease in anabolic activity might be a major determinant of decreased rod outer segment length in treated mice [5]. Further investigation into RPE metabolism in treated and untreated RP retinas is needed to advance our understanding of how metabolic homeostasis is crucial to maintain a proper functioning system between RPE and photoreceptors.

## Funding

This work was supported by the 10.13039/501100001659German Research Foundation [Emmy Noether grant KO 5719/1–1], the 10.13039/501100002974Daimler and Benz Foundation to S.F.K, the 10.13039/501100001659German Research Foundation (289242253 - HA 6014/8-1 and KO 5719/3-1), and the Bayerische Forschungsstiftung (1597-23).

## CRediT authorship contribution statement

**Monika Ayten:** Writing – original draft, Investigation, Conceptualization. **Nundehui Díaz-Lezama:** Data curation. **Hanaa Ghanawi:** Data curation. **Felia C. Haffelder:** Data curation. **Jacqueline Kajtna:** Methodology. **Tobias Straub:** Visualization, Software. **Marco Borso:** Investigation. **Axel Imhof:** Investigation. **Stefanie M. Hauck:** Investigation. **Susanne F. Koch:** Writing – review & editing.

## Declaration of competing interest

None.

## Data Availability

The data that has been used is confidential.
