# Supplementary material for: Metabolic plasticity in a Pde6bSTOP/STOP retinitis pigmentosa mouse model following rescue
Source: Mol Metab. 2024 Jul 19;88:101994. doi: 10.1016/j.molmet.2024.101994 (PMC11362769; doi:10.1016/j.molmet.2024.101994)
Supplement: Multimedia component. 1 [file mmc1.docx]

**9 Supplemental Information**

**
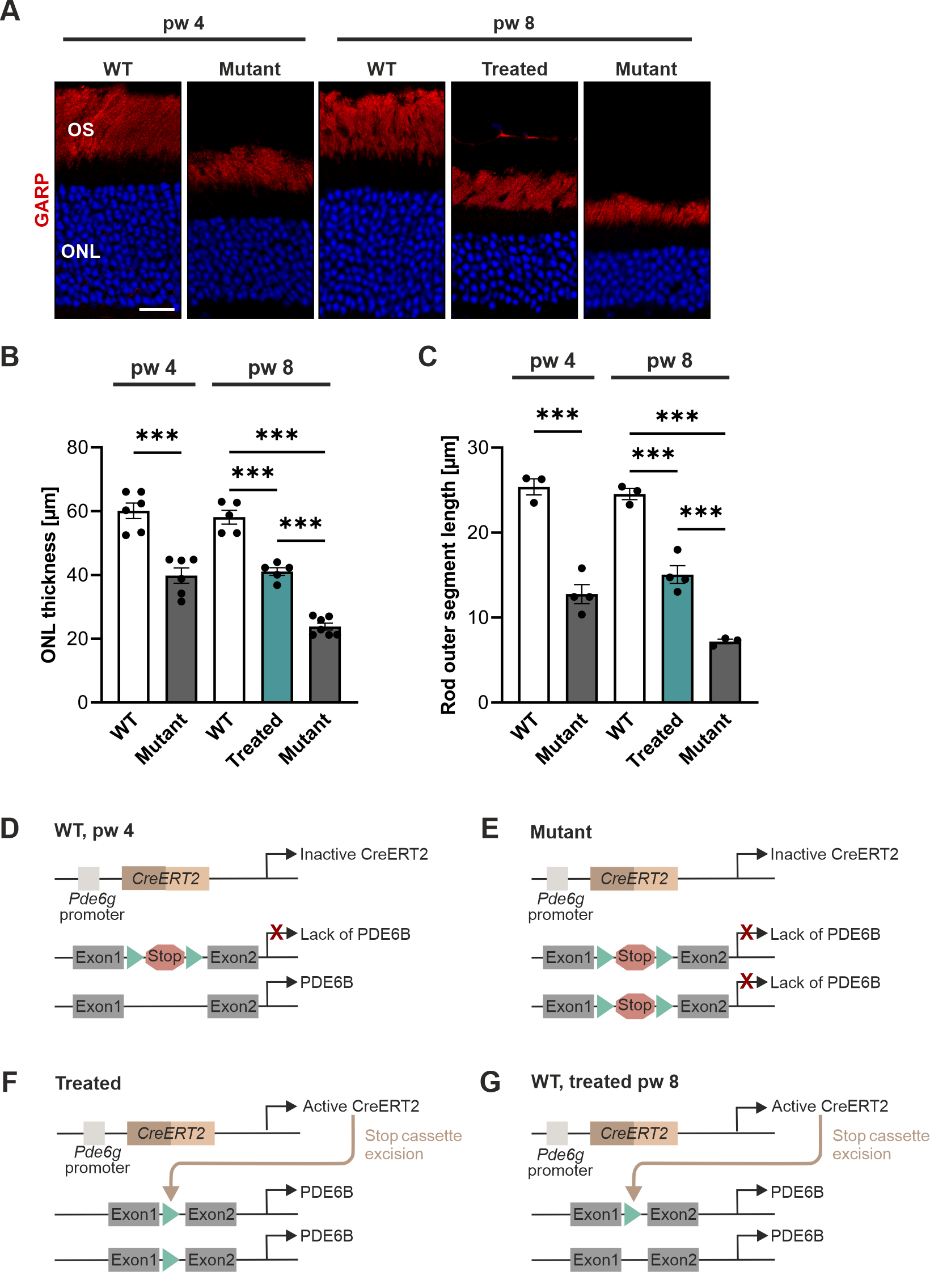
**

**Figure S1: Characterization of our mouse groups. (A)** Representative images of retinal sections immunostained for GARP and counterstained with Hoechst 33342. Scale bar, 15 µm. **(B)** Quantification of ONL thickness. **(C)** Quantification of rod OS length. **(B, C)** Data, presented as mean ± SEM, were compared by ANOVA. *** P ≤ 0.001. ONL, outer nuclear layer; OS, outer segment. **(D-G)** Genomic representation of our mouse groups. **(D)** *Pde6b^STOP/WT^* mice have 1 functional *Pde6b* allele; the second allele contains a floxed stop cassette inserted into intron 1 of the *Pde6b* gene. The tamoxifen-inducible CreERT2 recombinase is under the control of rod-specific Pde6g promoter and inactive. The mice were analyzed at 4 weeks of age (referred as WT, pW4) **(E*)*** *Pde6b^STOP/STOP^* mice contain a floxed stop cassette in both *Pde6b* alleles, which prevents PDE6B expression. The tamoxifen-inducible CreERT2 recombinase is under the control of rod-specific Pde6g promoter and inactive. The mice were analyzed at 4 weeks or 8 weeks of age (referred as mutant, pW4 and mutant, pW8) **(F)** *Pde6b^STOP/STOP^* mice were tamoxifen injected at 4 weeks of age to activate CreERT2 recombinase, which splices out the stop cassette, leading to PDE6B expression. Treated mice were analyzed at 8 weeks of age (referred as treated, pW8). **(G)** *Pde6b^STOP/WT^* mice were tamoxifen injected at 4 weeks of age to activate CreERT2 recombinase. The mice were analyzed at 8 weeks of age (referred as WT, pW8).


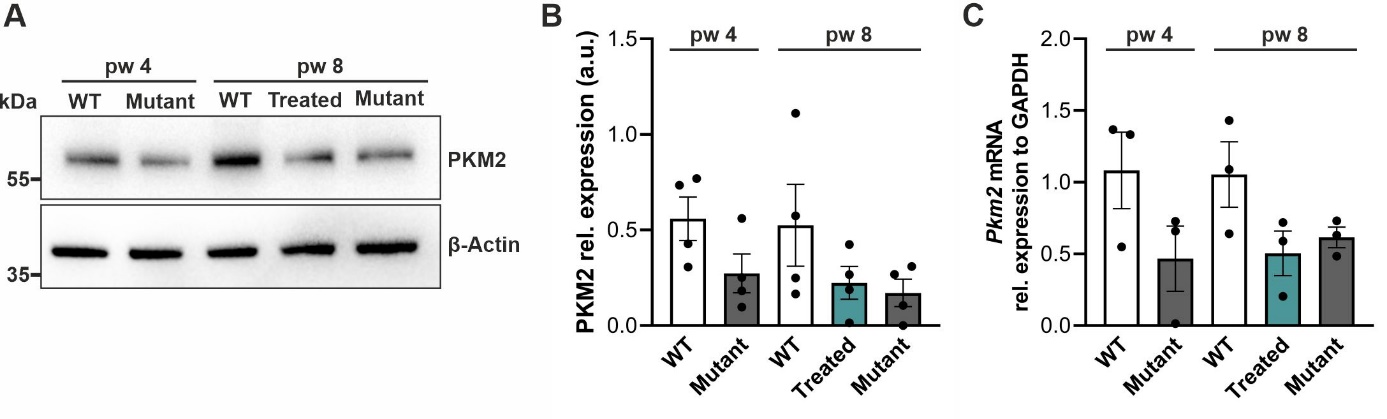


**Figure S2: Expression of PKM2 in treated and mutant retinas. (A)** Representative PKM2 immunoblot of retinal lysates. β-Actin was used as a loading control. **(B)** Quantitative analysis of PKM2 immunoblots. **(C)** Quantitative analysis of *Pkm2* mRNA by qRT-PCR.
